# Warpage Optimisation Using Recycled Polycar-bonates (PC) on Front Panel Housing

**DOI:** 10.3390/ma14061416

**Published:** 2021-03-15

**Authors:** Nur Aisyah Miza Ahmad Tamizi, Shayfull Zamree Abd Rahim, Abdellah El-hadj Abdellah, Mohd Mustafa Al Bakri Abdullah, Marcin Nabiałek, Jerzy J. Wysłocki, Bartłomiej Jeż, Paweł Palutkiewicz, Rozyanty Abdul Rahman, Mohd Nasir Mat Saad, Mohd Fathullah Ghazli

**Affiliations:** 1 Faculty of Mechanical Engineering Technology, Universiti Malaysia Perlis, Pauh Putra Main Campus, Arau 02600, Perlis, Malaysia; aisyahmiza94@gmail.com (N.A.M.A.T.); nasirsaad@unimap.edu.my (M.N.M.S.); fathullah@unimap.edu.my (M.F.G.); 2 Center of Excellence Geopolymer and Green Technology (CEGeoGTech), Universiti Malaysia Perlis, Kangar 01000, Perlis, Malaysia; mustafa_albakri@unimap.edu.my (M.M.A.B.A.); rozyanty@unimap.edu.my (R.A.R.); 3 Laboratory of Mechanics, Physics and Mathematical Modelling (LMP2M), University of Medea, Medea 26000, Algeria; lmp2m_cum@yahoo.fr; 4 Faculty of Chemical Engineering Technology, Universiti Malaysia Perlis, Pauh Putra Main Campus, Arau 02600, Perlis, Malaysia; 5 Department of Physics, Faculty of Processing Engineering and Materials Technology, Częstochowa University of Technology, 42-200 Częstochowa, Poland; nmarcell@wp.pl (M.N.); wyslocki.jerzy@wip.pcz.pl (J.J.W.); bartek199.91@o2.pl (B.J.); 6 Department of Polymer Processing, Faculty of Mechanical Engineering and Computer Science, Częstochowa University of Technology, 42-200 Częstochowa, Poland; palutkiewicz@ipp.pcz.pl

**Keywords:** injection moulding process, response surface methodology, genetic algorithms, warpage, recycle material

## Abstract

Many studies have been done using recycled waste materials to minimise environmental problems. It is a great opportunity to explore mechanical recycling and the use of recycled and virgin blend as a material to produce new products with minimum defects. In this study, appropriate processing parameters were considered to mould the front panel housing part using R0% (virgin), R30% (30% virgin: 70% recycled), R40% (40% virgin: 60% recycled) and R50% (50% virgin: 50% recycled) of Polycarbonate (PC). The manufacturing ability and quality during preliminary stage can be predicted through simulation analysis using Autodesk Moldflow Insight 2012 software. The recommended processing parameters and values of warpage in x and y directions can also be obtained using this software. No value of warpage was obtained from simulation studies for x direction on the front panel housing. Therefore, this study only focused on reducing the warpage in the y direction. Response Surface Methodology (RSM) and Genetic Algorithm (GA) optimisation methods were used to find the optimal processing parameters. As the results, the optimal ratio of recycled PC material was found to be R30%, followed by R40% and R50% materials using RSM and GA methods as compared to the average value of warpage on the moulded part using R0%. The most influential processing parameter that contributed to warpage defect was packing pressure for all materials used in this study.

## 1. Introduction

Presently, there are many scrap materials produced by moulding industries such as rejected parts and gating or feeding systems [1]. Other than the increase in plastic price due to the increase in oil price, the industrial sector also demands recycled plastic usage in order to reduce production costs [2]. A great amount of plastic waste occurs due to the increasing consumption of plastics all over the world [3]. The recycling of polymers and polymer composites has become a serious issue that had gained popularity among researchers in the 21st century [4,5]. There are many socioeconomical, political and technological drivers that may cause this issue to become more prominent in the near future [5,6].

Waste plastics are an untapped source of recovery for useful polymers. As an example, the demand for Polycarbonate (PC) in the year 2010 was approximately 3.4 million tons, with annual growth levels of approximately 6% [7,8]. Furthermore, PC is a thermoplastic polymer that has high chemical resistance with high temperatures and mechanical impact. PC is included in the carbonates group but is more expensive than other polymers that are commonly found in waste. On the other hand, in order to minimise environmental problems, several studies have also been conducted using recycled wastes of various thermoplastic materials [7,8,9,10,11,12,13].

For instance, Rahimi et al. [14], studied shrinkage, mechanical properties and structure of recycled Acrylonitrile Butadiene Styrene (ABS) by investigating the effect of reprocessing virgin material five times, and the results showed that the best mechanical properties of the moulded parts were achieved by using a 20% mixture of recycled and virgin materials, while the minimum shrinkage was obtained when using 50% mixture of recycled and virgin materials. Bhattacharya and Bepari [15] investigated the feasibility of Polypropylene (PP) recyclability in injection moulding based on a Gray Relational Analysis (GRA) and found that the injection speed is the most significant processing factor followed by the injection pressure, virgin-to-recycled ratio and lastly injection temperature. The optimal setting of processing parameters for recycled High Density Polyethylene (HDPE) using the Taguchi method was explored, and the results showed that any changes i processing parameters will eventually change or degrade the performance of the shrinkage, tensile strength and flexural strength of moulded parts produced. The optimisation methods used were able to generate an optimal setting of processing parameters to mould [16,17] the parts using virgin and recycled material in the injection moulding process [1,18,19].

Based on a review that had been done, many researchers defined warpage as a defect that affects the quality of the product [20,21,22,23,24,25]. the warpage defect can be minimised by determining an appropriate setting of processing parameters obtained from many beneficial optimisation methods. Therefore, the selection of an appropriate plastic material to fulfil the requirement of product specifications must be critically evaluated by the designer and manufacturer.

A few researchers used an optimisation method to reduce the defect on the moulded parts produced in injection moulding process by determining an optimal setting of processing parameters [26,27]. Nasir et al. [28] studied the effects of two types of gating systems using Autodesk Moldflow Insight (AMI) simulation software to analyse the shrinkage on parts produced in the injection moulding process. High packing pressure resulted in lower shrinkage, and it was also found that a single gate (0.3885%) gave a better result compared to the dual gates (0.4981%). Furthermore, Ozcelik and Erzurumlu [22] investigated the ways to minimise warpage on a plastic cover part by using integrated finite element analysis (FEA), statistical Design of Experiment (DOE), RSM and Genetic Algorithm (GA). The warpage of the part model was reduced by 40.4% (from 0.0977 mm to 0.0582 mm) using the GA optimisation method. Kurtaran and Erzurumlu [29] evaluated GA and RSM optimisation methods in part to optimise the value of warpage, and their results showed that warpage was decreased from 2.47 mm to 1.32 mm (46%) after optimisation using the GA method. It was also reported that the most significant factors influencing the warpage on the bus ceiling lamp were packing pressure (37.39%) followed by mould temperature (31.35%), melt temperature (26.94%), packing time (3.65%) and cooling time (0.6%).

Yin et al. [25] explored a hybrid optimising method in a plastic injection moulding process. This study combined a Back Propagation Neural Network (BPNN) method with an intelligent global optimisation algorithm, namely GA. After the BPNN/GA method was implemented, the warpage and the clamp force of the part were optimised and compared to the recommended value of warpage obtained from AMI simulation software. The optimised value of warpage was reduced by 66.9% (from 3.307 mm to 1.092 mm) compared to the recommended warpage value. In addition, the clamp force was increased 24.1% (from 63.63 tons to 79.01 tons) as compared to the recommended value suggested by AMI.

The Taguchi method is widely used in moulding industries to improve the warpage on the moulded part produced. The best combination of processing parameters can be determined by using the Taguchi method [21,30,31,32,33]. Although the Taguchi method can recommend the best combination setting of processing parameters, it is not suitable to be used in determining the optimum setting of processing parameter for continuous value. The major disadvantage of the Taguchi method is that the obtained results only display relative performance values, and the processing parameters with the highest effect cannot be determined [34,35]. On the other hand, RSM method is able to create a predictive model that shows the relation between processing parameters and warpage. The optimisation using the RSM method was used to reduce the shrinkage of the moulded parts produced in injection moulding process by obtaining the optimum value of processing parameters [22]. In addition, GA is one of the non-classical optimisation methods that has been recently used to obtain the optimal conditions of processing parameters in the injection moulding process [22,36].

It can be seen that the warpage defect can be minimised by determining an appropriate setting of processing parameters obtained from many beneficial optimisation methods. Therefore, the selection of an appropriate plastic material to fulfil the requirement of product specifications must be critically evaluated by the designer and manufacturer. On the other hand, in order to minimise environmental problems, several studies have also been conducted using recycled waste materials [2,4,6,7,37,38,39]. Therefore, the use of recycled and virgin blend materials to produce new products through the injection moulding process, at the same time as minimising defects by employing optimisation approaches, is a good opportunity to be explored.

In order to achieve a “go green environment”, plastic waste needs to be recycled, but the defects occurring on the moulded parts produced in the injection moulding process using recycle materials are hard to be diminished. Therefore, in this study, the effect of mixing the recycled and virgin materials on the warpage of moulded part produced is investigated. The optimisation methods are used to evaluate the optimum quality of moulded parts produced using optimal setting of processing parameters.

The results obtained will become a clear reference for the moulding industries to produce a new product using recycled materials with acceptable quality, which helps to increase company profits and minimize environmental waste.

## 2. Methodology

### 2.1. Finite Element Analysis Setup

**Figure 1:**
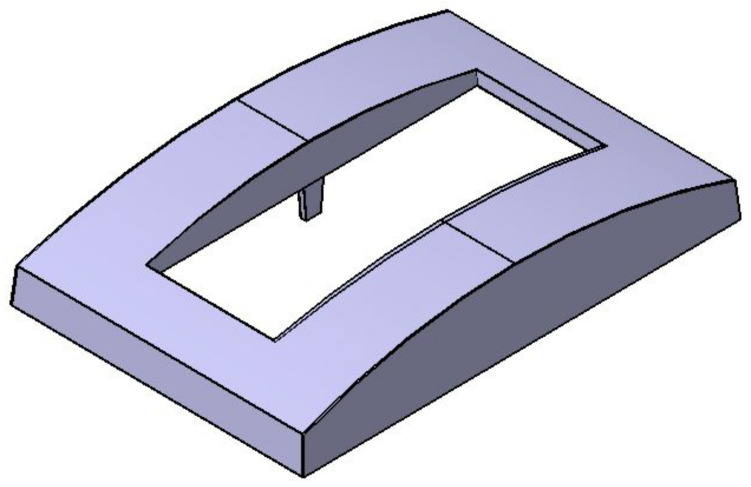
3D model of front panel housing.

First, simulation studies were conducted using Moldflow simulation software (Autodesk Moldflow Insight 2012, Moldflow, Melbourne, Australia) to analyse the flow of molten plastic material into the cavities [40]. The front panel housing with 2.5 mm thickness made from PC Panlite L1225LM manufactured by Teijin Kasei Malaysia Sdn, Bhd., Kuala Lumpur, Malaysia was used for the case study. The front panel housing with a curvature shape was selected to represent the current market trend of the product design as shown in Figure 1, while PC was chosen as the material to mould the front panel housing due to high demand and application of this type of plastic in industries today [4,7], which provides a good opportunity to explore mechanical recycling and the use of recycled and virgin blend materials as the materials to produce a new product with minimal defect. Thus, the warpage defect of recycled ratio on part produced using 30%, 40% and 50% recycled materials blended with 70%, 60% and 50% of virgin materials, respectively, were investigated and compared to the 100% virgin material. In addition, simulation studies were only performed for the 100% virgin material due to the constraint where the material properties for recycled materials are not available in the simulation software. However, all recycled materials blended with 100%, 70%, 60% and 50% of virgin materials were used to mould the parts in experimental works. Feeding systems, including sprue, runner and gate, were designed in AMI 2012 simulation software (Autodesk Moldflow Insight 2012, Moldflow, Melbourne, Australia) based on mould design guidelines [41,42]. This analysis was conducted to obtain an appropriate setting of processing parameters and also to predict the warpage on the front panel housing in x and y directions. Then, optimisation methods such as RSM and Artificial Intelligent methods integrated with simulation analysis were used to minimise the value of warpage on the moulded part produced.

### 2.2. Response Surface Methodology (RSM)

**Figure 2:**
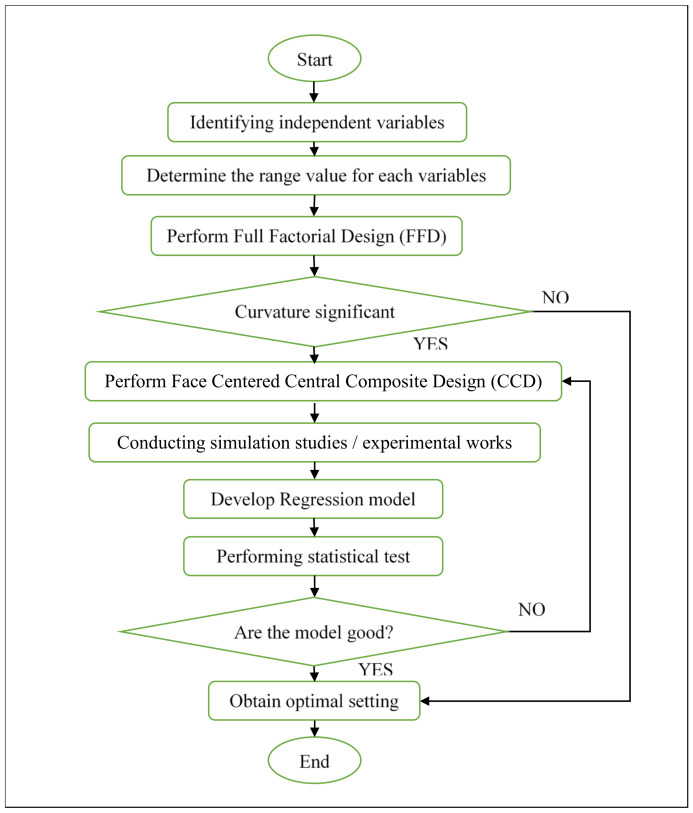
Methodology flowchart of RSM.

A predictive model was created using the RSM method, which shows the relation between processing parameters and warpage. In this study, the application of RSM and GA optimisation methods were used to determine the appropriate setting of processing parameters in order to minimise the warpage defect on the moulded parts. The values of warpage obtained in simulation studies and experimental work through RSM were compared with the value of warpage moulded through the GA method. Figure 2 shows the methodology flowchart to optimise the warpage defect on front panel housing using the RSM method.

#### 2.2.1. Design of Experiment Setup

Warpage that occurred on the moulded parts produced was analysed using these two levels (2^4^) Full Factorial Design (FFD). In addition, 2^4^ factorial design was used in this study due to the four processing parameters selected as variable parameters [43]. The range of selected processing parameters, which comprise melt temperature (°C), packing pressure (MPa), packing time (s) and cooling time (s) based on material specification and simulation analysis, are shown in Table 1. Therefore, 20 lists of experimental designs based on these four variables parameters and four centre points were generated. The significant curvature in FFD was best fit with the second order model in order to allow quadratic terms. Therefore, another 10 additional data of experimental runs were obtained and needed to be used as the setting of processing parameters to mould the front panel housing by performing Face-Centred Central Composite Design (CCD) [38]. An additional 10 runs were created within the range of minimum and maximum values when Face Centred CCD was selected. The cool (FEM) + fill + pack + warp analysis was performed using various settings of processing parameters in DOE (30 settings of processing parameters) to evaluate the value of warpage on front panel housing in x and y directions.

#### 2.2.2. RSM Regression Analysis

The relationship between variables and responses in this study is shown in the mathematical model obtained from regression analysis and was evaluated using the analysis of variance (ANOVA). The analysis of responses from all full factorial of experiments showed that the *p*-values of the curvature were below 0.05 [38]. The significant processing parameters and factor interactions were depicted through the ANOVA results. The insignificant processing parameters of the variables were eliminated by selecting the quadratic models. The selection process of backward elimination was suggested in a fit summary. Therefore, the significant model was remained in the hierarchy [38]. The polynomial regression model based on CCD analysis in RSM correlates the warpage with remaining inputs of processing parameters as stated in ANOVA, namely melt temperature (A), packing pressure (B), packing time (C) and cooling time (D). All of the mathematical equations obtained were used to run the analysis using the GA optimisation method.

### 2.3. Genetic Algorithm

**Figure 3:**
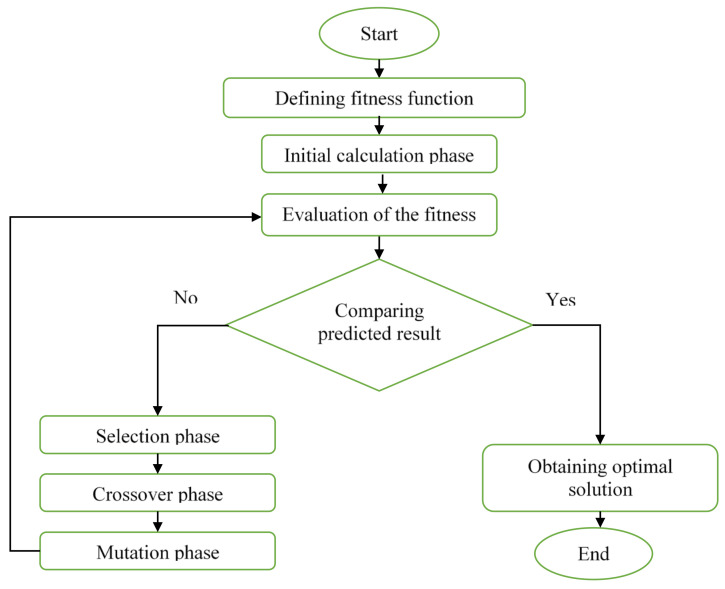
The methodology flowchart of genetic algorithm (GA).

The GA analysis was run using MATLAB programming language software (MATLAB R2014a, MathWorks, Natick, MA, USA) in order to execute the global optimum mathematical function obtained from RSM. The fitness function of mathematical model obtained from RSM analysis will be used to perform GA analysis. The main purpose of performing the GA analysis was to obtain the optimal setting of processing parameters to mould the front panel housing in experimental works and then compare the result of warpage on the front panel housing with RSM and recommended setting from AMI 2012 analysis. The methodology flowchart in conducting GA analysis is illustrated in Figure 3.

The mathematical equation obtained from the RSM using Design Expert software (Design Expert 7, Stat-Ease, Minneapolis, MN, USA) was used in GA analysis using MATLAB software as the fitness function. The initial calculation phase was set based on the selected variable parameters, and the bit number was calculated using Equation (1). The range of the variable parameters was set to create the solution space [39].

(1) $$2^{i\;-\;1}\;<\;(\;b\;\text{–}\;a\;)\;\times 10^{4}\;<\;2^{i}$$

where:

i—Bit number
b—Variable parameter maximum limit
a—Variable parameter minimum limit

The selection operator functions to determine the solutions that need to be preserved and reproduce, and which ones deserve to die out. The selection of genotypes (chromosomes) was based on the "fitness of the fittest," which implies that the best one would survive and create the new generation according to Darwinian theory. The primary objective of the recombination operator is to emphasize the good solutions and eliminate the bad solutions in a population while keeping the population size constant [44,45,46].

Crossover is the third basic process of GA operators and the process needs two genotypes called strings combined with their genetic material (bits), to produce a new offspring that possesses both of their characteristics. Two strings are picked from the mating pool randomly to crossover and create a genotype that might be better than both of the parents. The crossover is limited by the crossover rate and is not applied to the entire population strings [47,48].

The last basic GA operator is mutation. The mutation phase consists of changing each bit along the string. The mutation probability functions to modify one or more genes in a genotype from the initial solutions. A better solution can be determined from a new gene rate produced.

The evaluation of the fitness is done to determine the quality of each solution in the population. The fitness value can be used to perform the selection process, where the best ones are preserved and the others are discharged. The process is repeated until the best fitness is determined by a genotype and taken as an optimal solution [45,49].

### 2.4. Experimental Works

**Figure 4:**
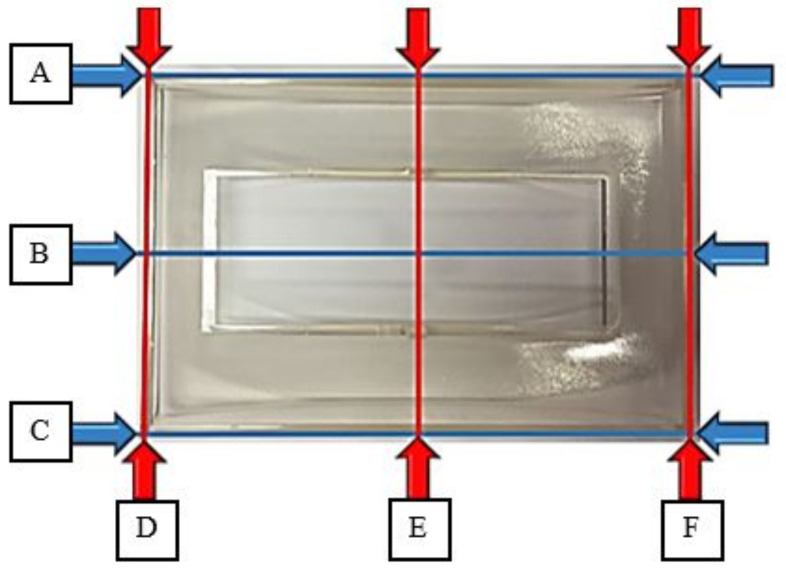
Measuring points on front panel housing to measure the warpage in y direction.

Experiments were conducted to evaluate the warpage on front panel housing based on the optimised processing parameters gained from RSM and GA analyses. The database of virgin (R0%) PC material can be used as a guidance to mould the front panel housing in experimental works by using R30%, R40% and R50% materials. This is due to the fact that the database for recycled material properties is not available in simulation software. The recycled material was prepared using a crusher machine (XC-GB20HP, Jiangmen Xiecheng Machinery Co., Ltd., Guangdong, China) to crush the rejected parts and gating system produced by using the virgin material. A sieve with a mesh size of 6 mm was integrated into the crusher machine. After crushing, the recycled materials that were larger than 6 mm were recycled again, while the recycled materials that were smaller than 6 mm dropped through the sieve into the collector bin placed at the lower part of crusher machine. Then, the digital weight scale was used to weigh the recycled material in order to prepare the required recycle material blends ratio. Next, the mixture of virgin and recycled materials was blended evenly using vertical mixer machine (XC-JB500L, Jiangmen Xiecheng Machinery Co., Ltd., Guangdong, China). The specimens of the front panel housing were moulded by using an injection molding machine (Nissei NEX1000, Nissei Plastic Indus-trial Co., Ltd., Minamijo, Japan). Then, the measuring point in x and y directions as shown in Figure 4 were measured using Coordinate Measuring Machine (CMM), (Mitutoyo Crysta-Plus M574, Mitutoyo Corporation, Kanagawa, Japan) after being stored in room temperature for 48 h according to International Standard Organisation (ISO) 291 [50]. Then, the maximum warpage in x and y directions was calculated using Equations (2) and (3).

(2) $$\mathrm{Maximum}\;\mathrm{warpage}\;\mathrm{in}\;x\;\mathrm{direction}=\mathrm{Maximum}\;\mathrm{value}\;\mathrm{of}\left(\frac{A-B}{2}\right)\mathrm{or}\left(\frac{C-B}{2}\right)$$

(3) $$\mathrm{Maximum}\;\mathrm{warpage}\;\mathrm{in}\;y\;\mathrm{direction}=\mathrm{Maximum}\;\mathrm{value}\;\mathrm{of}\left(\frac{D-E}{2}\right)\mathrm{or}\left(\frac{F-E}{2}\right)$$

## 3. Results and Discussion

### 3.1. Simulation Results

The recommended setting of processing parameters using PC material was identified by running the simulation analyses in AMI software. Table 2 shows the setting values of processing parameters obtained from simulation studies by using virgin material, while Table 3 presents the predicted value of warpage defect on front panel housing in x and y directions based on measurement dimensions A, B, C, D, E and F values obtained from simulation studies through AMI software. The minimum and maximum range of four selected processing parameters, namely are melt temperature, packing pressure, packing time and cooling time, are shown in Table 1. The range of melt temperature is obtained from material specification provided by material manufacturer, while packing pressure, packing time and cooling time were obtained from simulation analyses.

A simplified statistical analysis of ANOVA in two-level factorial for the warpage on front panel housing in y direction is tabulated in Table 4, while the statistical analysis of ANOVA for the warpage in the x direction could not be determined due to the zero values of warpage obtained. A significant effect is considered when the probability value is less than 0.05 (*p*-value < 0.05), while probability values greater than 0.05 (*p*-value > 0.05) indicate that it is an insignificant term [43]. All of the models are significant as the *p*-values are lesser than 0.05 [43]. An insignificant lack of fit obtained in the results of experimental works means a weak lack of fit. This indicates that a desirable property has been achieved, and there was no value of lack of fit found in simulation studies due to the same values of warpage obtained using the same values of processing parameters at the centre point of FFD. Therefore, the results of experimental works showed that the model fits the data well [51]. The R^2^ value for R0% (virgin) material in the simulation studies is 0.9857, while for R0% (virgin), R30%, R40% and R50% materials in experimental works, the values are 0.8011, 0.9107, 0.8455 and 0.8588 respectively. These show that the R^2^ value is almost 1 and is desirable. The difference between Pred R^2^ and Adj R^2^ values is less than 0.2, and this also represents that all models are reasonable [51]. In addition, all of these models have an adequate signal that can be used to navigate the design space due to the values being greater than 4 [51]. The significance of processing parameters that contribute the warpage defect was also analysed in the full factorial stage.

The DOE created 30 experimental runs, as shown in Table 5, which consist of 16 factorials, eight axial points and six centre points, yielding a quadratic effect towards the model [43]. Face-Centred CCD was selected in order to create the processing parameters within the range of minimum and maximum values. There was no significant curvature or warpage shown by the model in the front panel housing in the x direction. Thus, only the warpage that occurred in the y direction (from simulation studies) was taken to be measured and continued to be analysed in simulation studies and experimental works. All 30 settings of processing parameters used in the simulation study were used to set on the injection moulding machine in standard order to mould the front panel housing by using R0% (virgin), R30%, R40% and R50% materials. The values of warpage that occurred on the moulded part in the experimental works are shown in Table 5. CMM was used to measure the warpage on the moulded parts.

### 3.2. Analysis of Variance Result

The ANOVA results for virgin, R30%, R40% and R50% materials can be found in Table 6. The calculated value of F is greater than the value of F tabulated at the *p* = 0.05 level of significance, and this indicates that mathematical models obtained from regression analysis were significant [52]. It can be seen that R^2^ for R0% (virgin) material in the simulation studies is 0.9765, while for R0% (virgin), R30%, R40% and R50% materials in experimental works are 0.8149, 0.8222, 0.8156 and 0.8663, respectively. All R^2^ values are close to 1, which indicates that all the models fit the data.

### 3.3. RSM Regression Analysis Result

The summary of ANOVA results of CCD for simulation studies and experimental works is shown in Table 7.

All of the models are significant due to the *p*-values being below 0.05 [43]. An insignificant lack of fit obtained in the results of experimental works means a weak lack of fit, indicating that a desirable property has been achieved. Meanwhile, there was no value of lack of fit found in simulation studies due to the same values of warpage obtained using the same values of processing parameters at the centre point of FFD and CCD. Therefore, the results of experimental works showed that the model fits the data well [51]. The coefficient of determination (R^2^) values for R0% (virgin) material in simulation studies is 0.9765, while that for R0% (virgin), R30%, R40% and R50% materials in experimental works are 0.9765, 0.8149, 0.8222, 0.8156 and 0.8663, respectively. These show that the R^2^ value is close to 1 and hence is desirable. The difference between Predicted R^2^ (Pred R^2^) and Adjusted R^2^ (Adj R^2^) values is less than 0.2, and this also represents that all models are reasonable [51]. In addition, all of these models have an adequate signal that can be used to navigate the design space due to the values that are greater than 4 [51]. Equation (4) presents the relationship between the variable parameters and response of front panel housing moulded using R0% (virgin material) from simulation studies, while Equations (5)–(8) present the relationship between variables parameters and responses from experimental works using R0% (virgin), R30%, R40% and R50% recycled materials. All of these equations were used to run the analysis using the GA optimisation method.

Warpage in y direction using R0% (virgin material) (Simulation studies) = −1.20175 − 1.08674 × 10^−3^ × (A) + 1.0158 × 10^−3^ × (B) + 1.64481 × (C) − 4.32339 × 10^−3^ × (D) + 1.75439 × 10^3^ × (A) × (C) − 0.64635 × (C)^2^ (4)

Warpage in y direction using R0% (virgin material) (Experimental works) = 6.39468 − 4.93917 × 10^-3^ × (A) + 0.014541 × B + 1.77058 × (C) − 0.84758 × (D) − 5.60758 × 10^−5^ × (A) × (B) − 6.30263 × 10^3^ × (A) × (C) + 1.05285 × 10^−3^ × (A) × (D) + 0.016516 × (D) × (D) (5)

Warpage in y direction using R30% material (Experimental works) = 3.45178 + 4.51867 × 10^−4^ × A + 0.017389 × B + 2.12017 × C − 0.62456 × D − 6.50601 × 10^−5^ × A × B − 7.63596 × 10^3^ × A × C + 8.82944 × 10^−4^ × A × D + 0.011290 × D × D (6)

Warpage in y direction using R40% material (Experimental works) = 3.83903 + 3.81661 × 10^−3^ × A − 0.010588 × B + 1.99811 × C − 0.63615 × D − 7.32895 × 10^−3^ × A × C + 4.77626 × 10^−4^ × A × D + 5.63927 × 10^−4^ × B × D + 0.014262 × D × D (7)

Warpage in y direction using R50% material (Experimental works) = 5.91214 − 5.69484 × 10^−3^ × A + 0.016071 × B + 1.10949 × C − 0.70778 × D − 6.07583 × 10^−5^ × A × B − 4.09649 × 10^−3^ × A × C + 8.83268 × 10^−4^ × A × D + 0.013760 × D × D (8)

### 3.4. Analysis of Variance Result

An optimal setting of processing parameters was suggested for the regression models based on polynomial model in order to obtain minimum warpage on the front panel housing produced through RSM method. Table 8 shows the optimised setting of processing parameters and predicted warpage value from simulation studies and experimental works.

The optimum processing parameters and predicted values of warpage using R0% (virgin) material through GA method was obtained through simulation studies, while the predicted values of warpage on front panel housing moulded using R0% (virgin), R30%, R40% and R50% materials were obtained from experimental works. The optimal processing parameters obtained for simulation studies and experimental works through GA method are summarised in Table 9.

### 3.5. Experimental Work Results

The recommended settings of processing parameters obtained from AMI software are shown in Table 10 and were validated in experimental works. The results of warpage obtained using optimum processing parameters proposed by both optimisation methods (RSM and GA) were compared between simulation studies and experimental works. All of these results are presented in Table 11.

The warpage values on the front panel housing in the y direction from simulation studies were improved by 24.04% and 25.56% using RSM and GA methods, respectively. Meanwhile, the result of warpage values from experimental works improved by 2.87% and 0.61% using RSM and GA methods, respectively, as compared to recommended processing parameters suggested by AMI 2012. Based on the results of the analysis, it was found that there are minor differences between the results obtained from simulation studies and experimental works. This is due to the assumption that certain values of the processing parameters at the minimum and maximum melt temperature yield different results of warpage values. This indicates that, the simulation findings alone are not completely reliable and are less advantageous to the injection moulding industry. The results from simulation studies need to be validated in experimental works. Nevertheless, the results from simulation studies can be used as guidance for engineers in moulding industries.

The average results of warpage using RSM and GA methods for each material used in this study (R0%, R30%, R40% and R50%) are shown in Table 12. The percentage differences of R30%, R40% and R50% materials through the RSM method are 3.94%, 7.00% and 8.77%, respectively, while for those found using the GA method, the percentage differences are 3.29%, 5.76% and 7.14%, respectively, as compared to R0% material. Therefore, the results imply that the optimal ratio of the recycled materials to mould the front panel housing obtained from both RSM and GA optimisation methods through experimental works is R30%, followed by R40% and R50%. However, all predicted average warpage for all recycled material ratios are in the acceptable range, which is 0.4 mm of profile tolerances.

**Figure 5:**
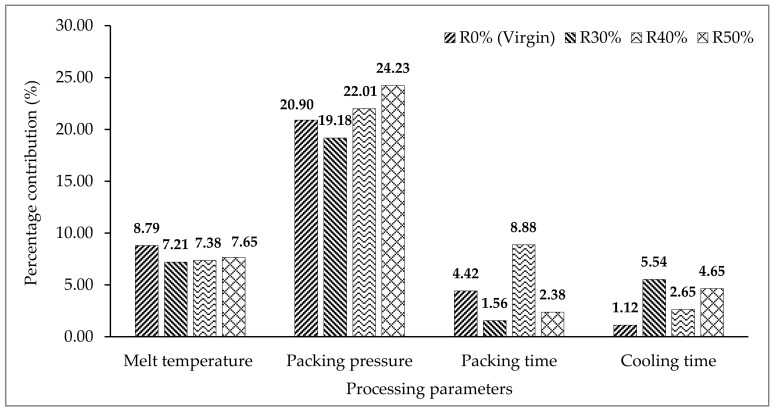
The percentage contribution of each processing parameters for virgin, R30%, R40% and R50%.

As can be seen in Figure 5, the most influential processing parameter that contributes to warpage defect is packing pressure when using R0% (virgin), R30%, R40% and R50% of PC material. The shrinkage phenomenon can be reduced by an appropriate setting of packing pressure in order to supply enough melt volume of molten material during the packing stage. An increment in the percentage of recycled material will increase the density of the melt polymer. Moreover, higher melt temperatures also contributed to a higher density and loosen the bonding between molecules. Therefore, an increase in polymer density as close as solid density required a high packing pressure to pack the molten material into mould cavities and reduce the warpage defect of the moulded parts produced. The second highest processing parameter that affects the warpage on the front panel housing moulded using R0% (virgin), R30% and R50% materials is melt temperature. On the other hand, melt temperature is the third processing parameter that influenced warpage on the front panel housing moulded using R40% material, while the second highest is packing time. However, packing time is the third processing parameter influencing warpage on the moulded part produced using R0% (virgin), while cooling time is the third processing parameter influencing warpage on parts produced using R30% and R50% materials. However, cooling time is less significant for the parts produced using R0% (virgin) and R40% materials, while packing time is less significant for the parts produced using R30% and R50% materials.

**Figure 6:**
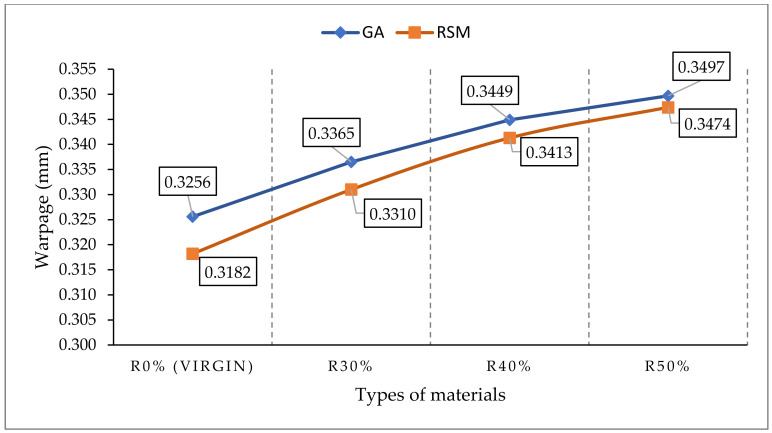
The warpage value for each type of materials used.

Table 13 shows the percentage difference of average warpage values for all materials used in this study (R0%, R30%, R40% and R50%) obtained through RSM and GA methods. The average warpage on the front panel housing moulded using R0% (virgin) material through RSM was improved by 2.29% (from 0.3256 mm to 0.3182 mm) as compared to the GA method. In addition, it can be seen that the average values of warpage in the y direction on the front panel housing moulded using R30%, R40% and R50% materials also improved by 1.65% (from 0.3365 mm to 0.3310 mm), 1.05% (from 0.3449 mm to 0.3413 mm) and 0.66% (from 0.3497 mm to 0.3474 mm), respectively, through RSM as compared to the GA method. As can be seen in Figure 6, the results showed that the RSM method offers a better value of average warpage on the front panel housing as compared to the GA method. However, the difference between both optimisation methods only showed a slight difference (less than 2.3%). The results of both optimisations (RSM and GA) showed an increment in warpage of values with the increase of the recycled ratio. Therefore, the lowest average warpage between the recycled material ratios was R30% followed by R40% and R50% for both optimisation methods (RSM and GA). Hence, the lower blend of virgin and recycled ratio offers a minimum warpage to mould the front panel housing part.

### 3.6. Validation Result of Simulation Studies with Experimental Works

The validation tests were performed by comparing the average warpage of five measured parts obtained from experimental works with the predicted warpage results from RSM and GA optimisation methods. The percentage errors were calculated for R0% (virgin), R30%, R40% and R50% materials using Equation (9) [25] in order to evaluate the validity of the simulated results. All of the percentage errors shown in Table 14 were below 15%, which is in good agreement and satisfies the validation of the experimental test [53].

(9) $$\mathrm{Percentage}\;\mathrm{error}\;\left(\%\right)\;=\left(\frac{\mathrm{Approximate}\;\mathrm{value}-\mathrm{Exact}\;\mathrm{value}}{\mathrm{Approximate}\;\mathrm{value}}\right)\;\times 100$$

## 4. Conclusions

The results from this study provide beneficial scientific knowledge and solutions to the moulding industries in improving the quality of moulded parts produced using a mixture of virgin and recycled blends of materials by employing optimisation methods such as RSM and GA. Therefore, from the obtained findings, the following can be concluded:

- The warpage in x direction was neglected as no warpage value was formed on front panel housing in simulation studies.
- Packing pressure was found to be the most influential processing parameter that contributed to the warpage defect for all mixture blend materials by 20.9%, 19.18%, 22.01% and 24.23% for R0%, R30%, R40% and R50%, respectively, in experimental works.
- The percentage difference of average values of warpage using RSM for R30%, R40% and R50% materials are 3.94%, 7.00% and 8.77%, respectively, as compared to the average value of warpage for R0% (virgin) material. Meanwhile, the percentage difference of average warpage using GA method for R30%, R40% and R50% materials are 3.29%, 5.76% and 7.14%, respectively, as compared to the average value of warpage moulded using R0% (virgin) material.
- The optimal ratio of recycled PC material using the RSM and GA method is R30% followed by R40% and R50% as compared to the value of average warpage that occurred on the front panel housing in the y direction moulded using R0% (virgin) material.
- RSM has been identified to be able to minimise the warpage on front panel housing moulded using R0%, R30%, R40% and R50% by 2.30%, 1.65%, 1.05% and 0.66%, respectively, based on validated warpage values that were measured by percentage difference as compared to the GA method in experimental works.
- All of the percentage errors of validation tests are below 15%, which is in good agreement and is satisfied by comparing the average warpage of experimental works with the predicted warpage results from RSM and GA optimisation methods.

Nonetheless, the results of average warpage on the front panel housing moulded using all ratios used in this study (R0%, R30%, R40% and R50%) are in an acceptable range of 0.4 mm (profile tolerances).

In the future, it will be beneficial to expand this kind of research to optimise the moulded part using hybrid methods such as hybrid GA-Particle Swarm Optimization(PSO); to replace the amorphous material (PC) with crystalline materials such as Polypropylene (PP), Polystyrene (PS); to study the effect of recycled material ratio; and to investigate the effect of recycled material ratio on the strength of the moulded parts produced.

## Figures and Tables

**Table 1:** Minimum and maximum values of processing parameters in two-level full factorial design of experiment.

Processing Parameter	Minimum	Maximum	Source
Melt temperature (°C)	270	300	Material specification
Packing pressure (MPa)	34.96	56.85	Fill + Pack analysis
Packing time (s)	1.58	1.77	Cool analysis
Cooling time (s)	15.45	18.02	Cool analysis

**Table 2:** Recommended setting of processing parameters.

Parameter Setting	Value
Melt temperature	285 °C
Mould temperature	95 °C
Cooling Inlet Temperature	80 °C
Fill time	2.64 s
V/P switch over	12 mm
Packing pressure	46.98 MPa
RAM position	62 mm
Cooling time	16.7 s
Packing time	1.5425 s

**Table 3:** Warpage on front panel housing from simulation using AMI 2012 software.

Directions	Nodes	Initial Dimension	Distance after	Warpage
Coordinate X direction	A	120 mm	119.5 mm	0
	B		119.5 mm	
	C		119.5 mm	
Coordinate Y direction	D	80 mm	79.62 mm	0.25
	E		79.12 mm	
	F		79.62 mm	

**Table 4:** Statistical analysis of ANOVA in two-level factorial for the warpage on the front panel housing moulded using virgin material in simulation studies and experimental works.

	Simulation Studies	Experimental Works			
	Virgin	Virgin	R30%	R40%	R50%
Model	<0.0001	0.0012	<0.0001	0.0010	<0.0001
Curvature	0.0272	0.0395	0.0032	0.0030	0.0038
Lack of fit	–	0.0940	0.4298	0.3978	0.2796
R^2^	0.9857	0.8011	0.9107	0.8455	0.8588
Adjusted R^2^	0.9802	0.7016	0.8538	0.7171	0.8045
Predicted R^2^	0.9637	0.5369	0.7169	0.5416	0.6931
Adequate R^2^	38.672	10.484	17.208	11.546	13.51

**Table 5:** Warpage results of DOE for front panel housing in y direction from simulation studies and experimental works.

Std order	Data Source	MT (°C)	PP (MPa)	PT (s)	CT (s)	Simulation Studies	Experimental Works			
						R0%	R0%	R30%	R40%	R50%
1	DOE	270	34.96	1.58	15.45	0.200	0.3841	0.3741	0.3801	0.3854
2		300	34.96	1.58	15.45	0.260	0.3647	0.3647	0.3627	0.3654
3		270	56.85	1.58	15.45	0.230	0.3684	0.3584	0.3534	0.3714
4		300	56.85	1.58	15.45	0.275	0.3128	0.3228	0.3248	0.3118
5		270	34.96	1.77	15.45	0.195	0.3837	0.3837	0.3837	0.3798
6		300	34.96	1.77	15.45	0.260	0.3459	0.3369	0.3469	0.3478
7		270	56.85	1.77	15.45	0.220	0.3680	0.3780	0.3380	0.3676
8		300	56.85	1.77	15.45	0.275	0.2630	0.2630	0.2830	0.2732
9		270	34.96	1.58	18.02	0.190	0.3647	0.3547	0.3537	0.3598
10		300	34.96	1.58	18.02	0.245	0.3931	0.3931	0.4031	0.3931
11		270	56.85	1.58	18.02	0.225	0.3128	0.3428	0.3438	0.3428
12		300	56.85	1.58	18.02	0.265	0.3758	0.3658	0.3598	0.3660
13		270	34.96	1.77	18.02	0.190	0.3551	0.3595	0.3595	0.3605
14		300	34.96	1.77	18.02	0.245	0.4021	0.3921	0.3321	0.3892
15		270	56.85	1.77	18.02	0.205	0.3630	0.3630	0.3630	0.3593
16		300	56.85	1.77	18.02	0.270	0.3315	0.3345	0.3345	0.3405
17	Centre	285	45.91	1.68	16.74	0.240	0.3220	0.3192	0.3092	0.3210
18		285	45.91	1.68	16.74	0.240	0.3281	0.3291	0.3241	0.3309
19		285	45.91	1.68	16.74	0.240	0.3320	0.3382	0.3332	0.3402
20		285	45.91	1.68	16.74	0.240	0.3411	0.3411	0.3311	0.3391
21	Axial	270	45.91	1.68	16.74	0.210	0.3572	0.3729	0.3629	0.3784
22		300	45.91	1.68	16.74	0.270	0.3464	0.3660	0.3653	0.3657
23		285	34.96	1.68	16.74	0.235	0.3456	0.3756	0.3853	0.3762
24		285	56.85	1.68	16.74	0.255	0.3598	0.3789	0.3801	0.3792
25		285	45.91	1.58	16.74	0.240	0.3428	0.3821	0.3761	0.3892
26		285	45.91	1.77	16.74	0.235	0.3630	0.3519	0.3618	0.3678
27		285	45.91	1.68	15.45	0.260	0.3765	0.3890	0.3890	0.4013
28		285	45.91	1.68	18.02	0.240	0.3906	0.3794	0.3864	0.3965
29	Centre	285	45.91	1.68	16.74	0.240	0.3460	0.3785	0.3676	0.3791
30		285	45.91	1.68	16.74	0.240	0.3490	0.3970	0.3690	0.3991

**Table 6:** ANOVA of response surface model for straight drilled cooling channels.

		Sum of Squares	DF	Mean Square	F-Value (calculated)	F-Value (tabulated)	R^2^
R0% of simulation studies	Model	0.017	6	0.002833	152.22	2.55	0.9765
	Residual	0.0004094	22	0.00001861			
	Cor Total	0.018	29				
R0% of experimental work	Model	0.019	8	0.002379	11.01	2.45	0.8149
	Residual	0.004324	20	0.0002162			
	Cor Total	0.024	29				
R30% of experimental work	Model	0.015	8	0.001918	11.56	2.45	0.8222
	Residual	0.003318	20	0.001659			
	Cor Total	0.023	29				
R40% of experimental work	Model	0.012	8	0.001518	11.06	2.45	0.8156
	Residual	0.002746	20	0.0001373			
	Cor Total	0.02	29				
R50% of experimental work	Model	0.015	8	0.001879	16.2	2.45	0.8663
	Residual	0.002321	20				
	Cor Total	0.024	29				
DF = Degrees of Freedom							
R^2^ = Coefficient of determination							
F-value (Calculated) = Calculation of Mean Square for the term divided by the Mean Square for the Residual.							
F-value (Tabulated) = Critical values for the F distribution (from Table)							

**Table 7:** Summary of ANOVA results of CCD for simulation studies and experimental works.

	Simulation Studies	Experimental Works			
	Virgin	Virgin	R30%	R40%	R50%
Model	<0.0001	<0.0001	<0.0001	<0.0001	<0.0001
Lack of fit	-	0.0602	0.3657	0.3272	0.5281
Standard Deviation, SD	0.0043	0.0150	0.0130	0.0120	0.0110
R^2^	0.9765	0.8149	0.8222	0.8156	0.8663
Adjusted R^2^	0.9701	0.7409	0.7511	0.7419	0.8128
Predicted R^2^	0.9554	0.4940	0.6012	0.5487	0.6978
Adequate R^2^	42.145	14.948	16.911	15.747	18.755

**Table 8:** The optimised processing parameters and results of predicted warpage on the front panel housing using Response Surface Methodology (RSM).

		Simulation Studies	Experimental Works			
		Virgin	Virgin	R30%	R40%	R50%
Processing parameters	Melt temperature (°C)	270	300	300	300	300
	Packing pressure (MPa)	35.18	56.85	56.85	56.85	56.85
	Packing time (s)	1.77	1.77	1.77	1.77	1.77
	Cooling time (s)	17.69	16.09	15.93	16.16	16.13
Output	Predicted warpage (mm)	0.1899	0.2906	0.2995	0.3050	0.3073

**Table 9:** The values of processing parameters and predicted warpage obtained through GA optimisation method.

		Simulation Study	Experimental Works			
		Virgin	Virgin	R30%	R40%	R50%
Processing parameters	Melt temperature (°C)	270	300	300	300	300
	Packing pressure (MPa)	34.96	56.85	56.85	56.85	56.85
	Packing time (s)	1.74	1.74	1.74	1.74	1.74
	Cooling time (s)	18.02	16.0973	16.0925	16.1500	16.0925
Output	Predicted warpage (mm)	0.1861	0.2824	0.3014	0.3053	0.3109

**Table 10:** The recommended settings obtained from AMI software.

	Recommended Setting
Melt temperature (°C)	285
Packing pressure (MPa)	46.98
Packing time(s)	1.54
Cooling time (s)	16.70
Predicted warpage (mm)	0.25
Validated warpage (mm)	0.3276

**Table 11:** Optimised results of warpage on front panel housing based on simulation studies and experimental works through RSM and GA methods.

		Simulation Studies		Experimental Works	
		RSM	GA	RSM	GA
Processingparameter	Melt temperature (°C)	270	270	300	300
	Packing pressure (MPa)	35.18	34.96	56.85	56.85
	Packing time (s)	1.77	1.74	1.77	1.74
	Cooling time (s)	17.69	18.02	16.09	16.0973
Warpage (mm)		0.1899	0.1861	0.3182	0.3256
Percentage Improvement (%)		24.04	25.56	2.87	0.61

**Table 12:** The results of warpage on front panel housing in y direction moulded using optimal processing parameters through RSM and GA methods.

		RSM				GA			
		R30%	R30%	R40%	R50%	R0%	R30%	R40%	R50%
Processing parameters	Melt temperature (°C)	300	300	300	300	300	300	300	300
	Packing pressure (MPa)	56.85	56.85	56.85	56.85	56.85	56.85	56.85	56.85
	Packing time (s)	1.77	1.77	1.77	1.77	1.74	1.74	1.74	1.74
	Cooling time (s)	16.09	15.93	16.16	16.13	16.0973	16.0925	16.1500	16.0925
	Average warpage (mm)	0.3182	0.3310	0.3413	0.3474	0.3256	0.3365	0.3449	0.3497
	Percentage Different (%)	-	3.94	7.00	8.77		3.29	5.76	7.14

**Table 13:** Percentage difference of warpage on front panel housing moulded using R0% (virgin), R30%, R40% and R50% through RSM and GA methods.

Recycle Ratios	Optimisation Methods	Processing Parameters				Output	
		Melt Temperature (°C)	Packing Pressure (MPa)	Packing Time (s)	Cooling Time (s)	Average Warpage (mm)	Percentage Different (%)
R0% (Virgin)	RSM	300	56.85	1.77	16.09	0.3182	2.29
	GA	300	56.85	1.74	16.10	0.3256	
R30%	RSM	300	56.85	1.77	15.93	0.3310	1.65
	GA	300	56.85	1.74	16.09	0.3365	
R40%	RSM	300	56.85	1.77	16.16	0.3413	1.05
	GA	300	56.85	1.74	16.15	0.3449	
R50%	RSM	300	56.85	1.77	16.13	0.3474	0.66
	GA	300	56.85	1.74	16.09	0.3497	

**Table 14:** Percentage errors of validation tests for experimental works.

		Melt Temperature (°C)	Packing Pressure (MPa)	Packing Time (s)	Cooling Time (s)	Predicted Warpage (mm)	Average Warpage (mm)	Percentage Error (%)
R0% (Virgin)	RSM	300	56.85	1.77	16.0900	0.2906	0.3182	8.67
	GA	300	56.85	1.77	16.0973	0.2824	0.3256	13.27
R30%	RSM	300	56.85	1.77	15.9300	0.2995	0.3310	9.52
	GA	300	56.85	1.77	16.0925	0.3014	0.3365	10.43
R40%	RSM	300	56.85	1.77	16.1600	0.3050	0.3413	10.64
	GA	300	56.85	1.77	16.1500	0.3053	0.3449	11.48
R50%	RSM	300	56.85	1.77	16.1300	0.3073	0.3474	11.54
	GA	300	56.85	1.77	16.0925	0.3109	0.3497	11.10

## Data Availability

Not applicable.

## References

[1] Abdullah J., Shanb L., Ismail H. (2016). Optimization of injection moulding process parameters for recycled high density polyethylene (rhdpe) using the Taguchi method. Int. J. Mech. Prod. Eng..

[2] Kuram E., Timur G., Ozcelik B., Yilmaz F. (2014). Influences of injection conditions on strength properties of recycled and virgin PBT/PC/ABS. Mater. Manuf. Process..

[3] Soliman O., Abdelfattah I., Ibrahim S. (2016). Environmental recycling of compact disc using industrial wastewater. Der Pharmacia Lettre.

[4] Vidakis N., Petousis M., Maniadi A., Koudoumas E., Vairis A., Kechagias J. (2020). Sustainable additive manufacturing: Mechanical response of Acrylonitrile-Butadiene-Styrene over multiple recycling processes. Sustainability.

[5] Krauklis A.E., Karl C.W., Gagani A.I., Jørgensen J.K. (2021). Composite material recycling technology—state-of-the-art and sustainable development for the 2020s. J. Compos. Sci..

[6] Rissman J., Bataille C., Masanet E., Aden N., Morrow III W.R., Zhou N., Elliott N., Dell R., Heeren N., Huckestein B. (2020). Technologies and policies to decarbonize global industry: Review and assessment of mitigation drivers through 2070. Appl. Energy..

[7] Achilias D.S., Antonakou E.V. (2013). Recent advances in Polycarbonate recycling: A review of degradation methods and their mechanisms. Waste Biomass Valori..

[8] Mengistu N., Koneru S.N., Reddy A.I., Koteswararao B. (2019). Design and development of hand operated injection moulding machine for manufacturing recycled plastic. Int. J. Adv. Manuf. Tech..

[9] Chandrasekaran S.R., Avasarala S., Murali D., Rajagopalan N., Sharma B.K. (2018). Materials and energy recovery from e-waste plastics. ACS Sustain. Chem. Eng..

[10] Kuram E., Ozcelik B., Yilmaz F., Timur G., Sahin Z.M. (2014). The effect of recycling number on the mechanical, chemical, thermal and rheological properties of PBT/PC/ABS ternary blends: With and without glass-fiber. Polym. Compos..

[11] De Leo V., Casiello M., Deluca G., Cotugno P., Catucci L., Nacci A., D’Accolti L. (2021). Concerning Synthesis of new biobased polycarbonates with curcumin in replacement of bisphenol a and recycled diphenyl carbonate as example of circular economy. Polymers.

[12] Avolio R., Spina F., Gentile G., Cocca M., Avella M., Carfagna C., Tealdo G., Errico M.E. (2019). Recycling polyethylene-rich plastic waste from landfill reclamation: Toward an enhanced landfill-mining approach. Polymers.

[13] Galve J.E., Elduque D., Pina C., Clavería I., Acero R., Fernández Á., Javierre C. (2019). Dimensional stability and process capability of an industrial component injected with recycled polypropylene. Polymers.

[14] Rahimi M., Esfahanian M., Moradi M. (2014). Effect of reprocessing on shrinkage and mechanical properties of ABS and investigating the proper blend of virgin and recycled ABS in injection moulding. J. Mater. Process. Technol..

[15] Bhattacharya D., Bepari B. (2014). Feasibility study of recycled polypropylene through multi response optimisation of injection moulding parameters using grey relational analysis. Procedia Eng..

[16] Guan N., Hu C., Guan L., Zhang W., Yun H., Hu X. (2020). A Process optimization and performance study of environmentally friendly waste newspaper/polypropylene film layered composites. Materials.

[17] Wüst P., Edelmann A., Hellmann R. (2020). Areal surface roughness optimization of maraging steel parts produced by hybrid additive manufacturing. Materials.

[18] Mizamzul N.M., Kamaruddin S. (2011). Optimisation of mechanical properties of recycled plastic products via optimal processing parameters using the Taguchi method. J. Mater. Process. Technol..

[19] Scaffaro R., Botta L., Benedetto G.D. (2012). Physical properties of virgin-recycled ABS blends: Effect of post-consumer content and of reprocessing cycles. Eur. Polym. J..

[20] Chen W.C., Kurniawan D. (2014). Process parameters optimisation for multiple quality characteristics in plastic injection moulding using Taguchi method, BPNN, GA, and hybrid PSO-GA. Int. J. Precis. Eng. Manuf..

[21] Ozcelik B., Sonat I. (2019). Warpage and structural analysis of thin shell plastic in the plastic injection moulding. Mater. Des..

[22] Ozcelik B., Erzurumlu T. (2005). Determination of effecting dimensional parameters on warpage of thin shell plastic parts using integrated Response Surface Method and Genetic Algorithm. Int. Commun. Heat Mass Transf..

[23] Shi H., Gao Y., Wang X. (2010). Optimization of injection moulding process parameters using integrated Artificial Neural Network model and expected improvement function method. J. Mater. Process. Technol..

[24] Sun B., Wu Z., Gu B. Optimisation of injection moulding process parameters based on Response Surface Methodology and Genetic Algorithm, computer engineering and technology. Proceedings of the 2nd International Conference on Computer Engineering and Technology.

[25] Yin F., Mao H., Hua L. (2011). A hybrid of back propagation Neural Network and Genetic Algorithm for optimisation of injection moulding process parameters. Mater. Des..

[26] Chiang K.T., Chang F.P. (2007). Analysis of shrinkage and warpage in an injection-moulded part with a thin shell feature using the Response Surface Methodology. Int. J. Adv. Manuf..

[27] Ozcelik B., Erzurumlu T. (2006). Comparison of the warpage optimisation in the plastic injection moulding using ANOVA, Neural Network model and Genetic Algorithm. J. Mater. Process. Technol..

[28] Nasir S.M., Ismail K.A., Shayfull Z. (2016). Application of RSM to optimise moulding conditions for minimizing shrinkage in thermoplastic processing. Key Eng. Mater..

[29] Kurtaran H., Erzurumlu T. (2006). Efficient warpage optimisation of thin shell plastic parts using Response Surface Methodology and Genetic Algorithm. Int. J. Adv. Manuf. Technol..

[30] Oktem H. (2012). Optimum process conditions on shrinkage of an injected moulded part of DVD-ROM cover using Taguchi robust method. Int. J. Adv. Manuf. Technol..

[31] Altan M. (2010). Reducing shrinkage in injection mouldings via the Taguchi, ANOVA and Neural Network methods. Mater. Des..

[32] Sun C.H., Chen J.H., Sheu L.J. (2010). Quality control of the injection moulding process using an EWMA predictor and minimum–variance controller. Int. J. Adv. Manuf. Technol..

[33] Ozcelik B., Ozbay A., Demirbas E. (2010). Influence of injection parameters and mould materials on mechanical properties of ABS in plastic injection moulding. Int. Commun. Heat Mass Transf..

[34] Chen W.C., Nguyen M.H., Chiou H.S. (2016). Optimisation of the plastic injection moulding process using Taguchi method, RSM, and GA. Int. J. Adv. Manuf. Technol..

[35] Maghsoodloo S., Ozdemir G., Jordan V., Huang C.H. (2004). Strengths and limitations of Taguchi’s contributions to quality, manufacturing and process engineering. J. Manuf. Syst..

[36] Puertas I., Luis C.J. (2004). A study of optimisation of machining parameters for electrical discharge machining of boron carbide. Mater. Manuf. Process..

[37] Jaya H., Omar M.F., Akil H.M., Ahmad Z.A., Zulkepli N.N., Abdullah M.M.A., Sandu I.G., Vizureanu P. (2016). Effect of surface modification on sawdust reinforced High Density Polyethylene composites under a wide range of strain rates. Mater. Plast..

[38] Popita G.E., Rosu C., Manciula D., Corbu O., Popovici A., Nemes O., Sandu A.V., Proorocu M., Dan S.B. (2016). Industrial tanned leather waste embedded in modern composite materials. Mater. Plast..

[39] Maraveas C. (2020). Production of sustainable construction materials using agro-wastes. Materials.

[40] Zink B., Szabó F., Hatos I., Suplicz A., Kovács N.K., Hargitai H., Tábi T., Kovács J.G. (2017). Enhanced injection molding simulation of advanced injection molds. Polymers.

[41] Mok S.L., Kwong C.K., Lau W.S. (2001). A hybrid Neural Network and Genetic Algorithm approach to the determination of initial process parameters for injection moulding. Int. J. Adv. Manuf. Technol..

[42] Shoemaker J. (2006). Moldflow Design Guide: A Resource for Plastics Engineers.

[43] Montgomery D.C. (2009). Design and Analysis of Experiments.

[44] Vijayan D., Seshagiri V.R. (2018). Comparison of Response Surface Methodology and Genetic Algorithm in parameter optimization of laser welding process. Appl. Math. Inf. Sci..

[45] Huang M.L., Hung Y.H., Yang Z.S. (2016). Validation of a method using Taguchi, Response Surface, Neural Network and Genetic Algorithm. Measurement.

[46] Mishra A. Analysis of the effect of elite count on the behavior of genetic algorithms: A perspective. Proceedings of the 2017 IEEE 7th International Advance Computing Conference.

[47] Kannan G., Noorul A.H., Devika M. (2009). Analysis of closed loop supply chain using genetic algorithm and particle swarm optimisation. Int. J. Prod. Res..

[48] Alvarez M.J., Ilzarbe L., Viles E., Tanco M. (2009). The use of Genetic Algorithms in Response Surface Methodology. Qual. Technol. Quant. Manag..

[49] Vijayan D., Seshagiri V.R. (2018). A parametric optimization of FSW process using RSM based grey relational analysis approach. Int. Rev. Mech. Eng..

[50] ISO 291 Plastics–Standard atmospheres for conditioning and testing. https://www.iso.org/obp/ui/#iso:std:iso:291:ed-4:v1:en.

[51] Stat-Ease I. (2005). Design-Expert [Computer Software].

[52] Livingstone D.J., Salt D.W. (2005). A practical guide to scientific data analysis. J. Med. Chem..

[53] Desai K.M., Survase S.A., Saudagar P.S., Lele S.S., Singhal R.S. (2008). Comparison of Artificial Neural Network (ANN) and Response Surface Methodology (RSM) in fermentation media optimization: Case study of fermentative production of scleroglucan. Biochem. Eng. J..

